# The Effect of Pomegranate Peel Extract on the Oxidative and Inflammatory Status in the Spleens of Rats with Metabolic Syndrome

**DOI:** 10.3390/ijms252212253

**Published:** 2024-11-14

**Authors:** Alina Rak-Pasikowska, Kornela Hałucha, Marta Kamińska, Joanna Niewiadomska, Agnieszka Noszczyk-Nowak, Iwona Bil-Lula

**Affiliations:** 1 Division of Clinical Chemistry and Laboratory Haematology, Department of Medical Laboratory Diagnostics, Faculty of Pharmacy, Wroclaw Medical University, Borowska 211A, 50-556 Wroclaw, Poland; k.halucha@umw.edu.pl (K.H.); iwona.bil-lula@umw.edu.pl (I.B.-L.); 2 Lower Silesian Oncology, Pulmonology and Hematology Center, 12 Hirszfeld Square, 53-413 Wrocław, Poland; 3 Department of Internal Medicine and Clinic of Diseases of Horses, Dogs and Cats, Faculty of Veterinary Medicine, University of Environmental and Life Sciences, Grunwaldzki Square 47, 50-375 Wrocław, Poland; joanna.niewiadomska@upwr.edu.pl (J.N.); agnieszka.noszczyk-nowak@upwr.edu.pl (A.N.-N.)

**Keywords:** metabolic syndrome, syndrome X, punica granatum, polyphenols, oxidative stress, antioxidants, spleen, rats

## Abstract

Polyphenols have antioxidant and anti-inflammatory properties and maintain the immune system in balance; therefore, the aim of the study was to investigate the effect of polyphenols present in pomegranate peel extract on the spleens of rats with metabolic syndrome. The study objects were adult male Zucker Diabetic Fatty (ZDF-Leprfa/Crl, fa/fa) rats. The rats were divided into a control group (MetS) consisting of rats with metabolic syndrome and four study groups consisting of rats with metabolic syndrome (MetS + 100 mg and MetS + 200 mg) or healthy animals (H + 100 mg and H + 200 mg) receiving polyphenol extract at a dose of 100 mg or 200 mg/kg, respectively. Concentrations of IL-6, NF-κB, NFATc1, Cyt-C, TNFα, MMP-2, ROS/RNS, and MDA were measured; the activities of GPX, SOD, CAT, MMP-2, and MMP-9 were assessed; and the expression of the *BAX* and *BCL-2* genes was evaluated in homogenized spleens. In conclusion, pomegranate extract may lead to an increase in catalase and glutathione peroxidase activity. Additionally, it may have a reducing effect on the ROS/RNS level, leading to a reduction in the activity of SOD in the MetS groups with PPE administration. Moreover, the *BCL-2* gene showed lower expression in the MetS + 100 mg group compared to the H + 100 mg group, indicating that the balance between pro- and antiapoptotic factors of the BCL-2 family may be disrupted by the metabolic syndrome promoting the proapoptotic pathway.

## 1. Introduction

Recently, natural flavonoid compounds of plant origin have become increasingly popular due to their excellent pharmacological properties [1]. Polyphenols, as natural products found in large quantities in vegetables and fruits, have the ability to modulate antioxidant factors, scavenge free oxygen and nitrogen radicals, as well as control the formation of cellular redox transcription factors and maintain proper metabolic functions [2,3]. Polyphenols may also influence intercellular signaling, gene regulation, inflammatory enzyme activity, and receptor sensitivity [4,5,6]. Their beneficial effects largely depend on their bioavailability in the target tissue, as well as cellular distribution and metabolism after absorption [3,7]. In this context, there is currently great interest in studying the bioavailability and bioactivity of polyphenols and their role in modulating the redox state in vivo [8,9]. It has also been noticed that polyphenols have antiapoptotic, antihepatotoxic, antiallergic, and analgesic properties [1,10]. Resveratrol, a polyphenolic compound found in grapes, peanuts, and blueberries, has been proven to protect against cardiovascular diseases, various types of cancer, and ischemic injuries and to increase resistance to stress, as well as extend the lifespan of various organisms due to its anti-inflammatory, antioxidant, and antibacterial properties [11,12,13,14]. Baicalin, another flavonoid, was able to suppress nuclear factor kappa-light-chain-enhancer of activated B cells (NF-κB), a critical regulator of inflammation [15], which may prevent splenic immune impairment during infection.

Pomegranate (*Punica granatum* L.) has been used since ancient times and was treated as a valuable, traditional medicine in folk medicine [16,17]. Nowadays, different parts of the plant are being studied and their beneficial properties are described. Seeds, peel, juice, and leaves are rich in bioactive compounds such as flavonoids, phenolic acids, tannins, and anthocyanins [17,18] Pomegranate peel extract has been shown to be a more powerful antioxidant than pomegranate pulp extract. The total phenolic and flavonoid content was also higher in peel than in pulp [16]. According to the literature, pomegranate peels are rich in anthocyanin, ellagic acid, gallic acid, catechin, quercetin, rutin, kaempferol, cyaniding, punicalin, punicalagin, linoleic acid, chlorogenic acid, tannin, pelletierine alkaloids, and many other compounds [18]. Pomegranate peel extract contains various types of polyphenolic compounds, including punicalin, punicalagin and its isomers, ellagic acid derivatives, granatin A, and ellagitannin [19]. Pomegranate has been shown to have antidiabetic, anti-inflammatory, antiatherosclerotic, antimicrobial, antifungal, antihypertensive, anticancer, and antihyperlipidemic effects [20].

The spleen as the largest lymphatic organ, is a part of the immune system, playing a key role in the innate immune system and protecting the body against pathogen invasion and cell damage [1,21,22]. It modulates the differentiation and activation of inflammatory cells [23]. Nonalcoholic fatty liver disease, which is one of the most serious causes of liver disease worldwide, is associated with obesity and metabolic syndrome [24]. There are previous studies indicating an association between increased spleen size, obesity, and nonalcoholic fatty liver disease [25,26,27]. One possible explanation for this association is that the spleen may become enlarged as a result of chronic inflammation associated with insulin resistance and metabolic syndrome. This inflammation is caused by the overproduction of inflammatory mediators such as tumor necrosis factor-α (TNF-α) and IL-6, and a reduction in anti-inflammatory IL-10 [23,27,28]. Metabolic syndrome is becoming a universal problem associated with obesity, diabetes, and dyslipidemia [29]. Lipid accumulation causes low-grade inflammation resulting from an imbalance between pro-inflammatory and anti-inflammatory components of the immune system [30]. Metabolic syndrome (or syndrome X) significantly increases the risk of cardiovascular diseases and type 2 diabetes, which may ultimately result in premature death [31]. According to global data, with the spread of the “Western lifestyle”, the average BMI in both men and women and the prevalence of syndrome X are increasing. Data showed that almost 40% of people suffer from metabolic syndrome in the sixth decade of life [31,32,33]. A meta-analysis shows that the increasing association between syndrome X, and morbidity and mortality requires more intensive public health interventions [31], and lifestyle modifications, such as dietary changes, are an urgent global need.

Since polyphenols have anti-inflammatory and antioxidant effects and maintain the balance of the immune system, the aim of the study was to investigate the effect polyphenols, obtained in the form of pomegranate peel extract (PPE), on the spleens of rats with metabolic syndrome. We focused on the antioxidants as well as immune response and apoptosis pathway.

## 2. Results

### 2.1. Oxidative Status

**Figure 1:**
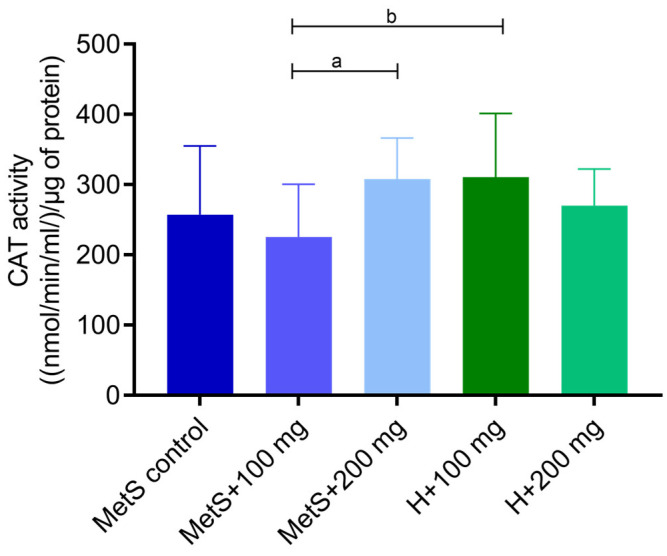
Catalase activity (CAT) in splenic tissue. MetS—control group of rats with mutation in the leptin receptor gene; MetS + 100 mg and MetS + 200 mg groups constituted animals with mutation and receiving polyphenol extract from pomegranate fruit peels at a dose of 100 mg/kg or 200 mg/kg, respectively; H + 100 mg and H + 200 mg—healthy animals without metabolic syndrome receiving polyphenol extract at a dose of 100 mg/kg or 200 mg/kg. a and b = *p* < 0.05. *n* = 6; Kruskal–Wallis test followed by Dunn test was used for comparison of MetS vs. MetS + 100 mg vs. MetS + 200 mg, and Mann–Whitney U test was used for MetS + 100 mg vs. H + 100 mg and for MetS + 200 mg vs. H + 200 mg comparisons.

**Figure 2:**
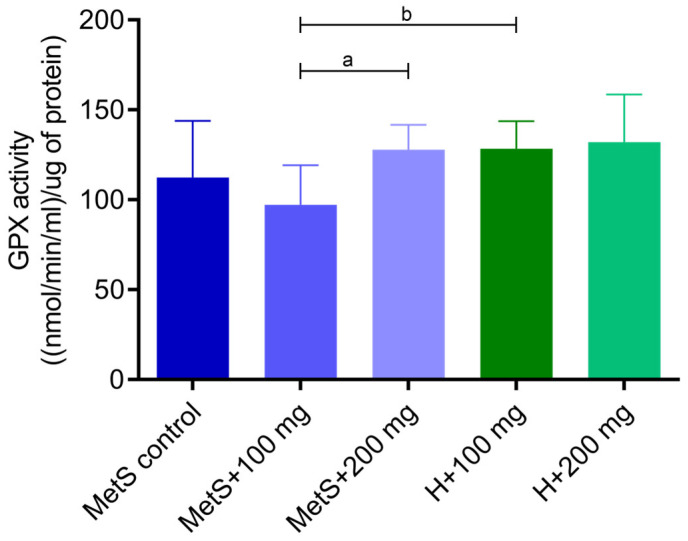
Glutathione peroxidase (GPX) activity in splenic tissue. MetS—control group of rats with mutation in the leptin receptor gene; MetS + 100 mg and MetS + 200 mg groups constituted animals with mutation and receiving polyphenol extract from pomegranate fruit peels at a dose of 100 mg/kg or 200 mg/kg, respectively; H + 100 mg and H + 200 mg—healthy animals without metabolic syndrome receiving polyphenol extract at a dose of 100 mg/kg or 200 mg/kg. a = *p* < 0.05, b = *p* < 0.01. *n* = 6; one-way ANOVA followed by Tukey’s test was used for comparison of MetS vs. MetS + 100 mg vs. MetS + 200 mg, and a t-test was used for MetS + 100 mg vs. H + 100 mg and for MetS + 200 mg vs. H + 200 mg comparisons.

Catalase (CAT) (Figure 1) and glutathione peroxidase (GPX) activities (Figure 2) were higher in splenic tissue from MetS rats receiving 200 mg/kg of the PPE compared to the group receiving 100 mg/kg of the extract. The “dose–response” effect in metabolic syndrome groups (MetS + 100 and MetS + 200 mg) was observed. Moreover, the GPX and the CAT activity were lower in the MetS + 100 mg group compared to the H + 100 mg group, but in groups receiving 200 mg of PPE, the activity in the MetS group and healthy objects was at the same level.

**Figure 3:**
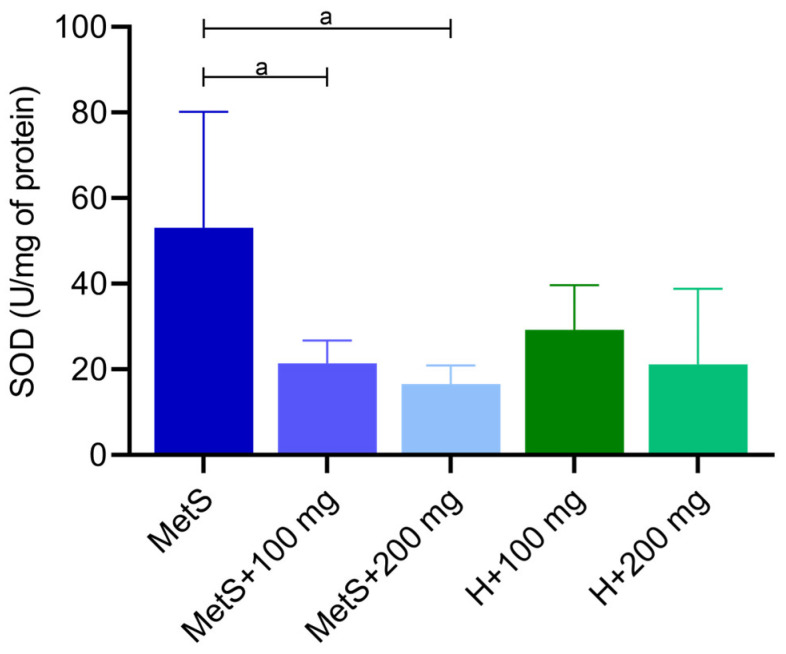
Superoxide dismutase (SOD) activity in splenic tissue. MetS—control group of rats with mutation in the leptin receptor gene; MetS + 100 mg and MetS + 200 mg groups constituted animals with mutation and receiving polyphenol extract from pomegranate fruit peels at a dose of 100 mg/kg or 200 mg/kg, respectively; H + 100 mg and H + 200 mg—healthy animals without metabolic syndrome receiving polyphenol extract at a dose of 100 mg/kg or 200 mg/kg. a = *p* < 0.01. *n* = 6; one-way ANOVA followed by Tukey’s test was used for comparison of MetS vs. MetS + 100 mg vs. MetS + 200 mg, and a t-test was used for MetS + 100 mg vs. H + 100 mg and for MetS + 200 mg vs. H + 200 mg comparisons.

**Figure 4:**
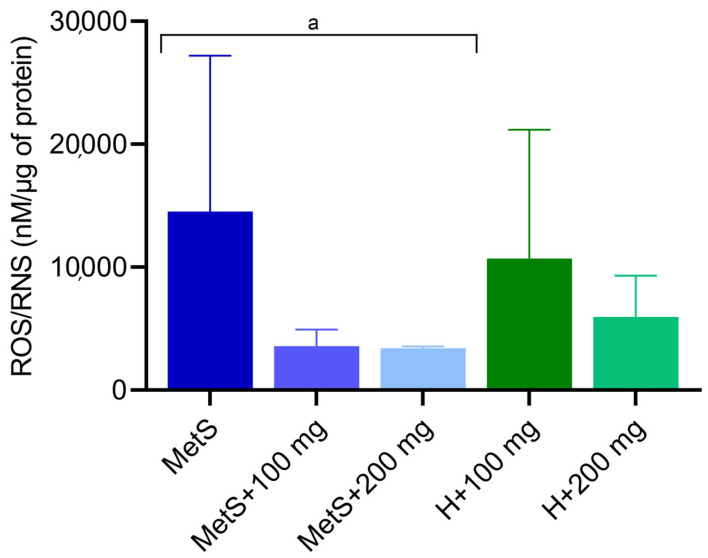
ROS/RNS concentration in splenic tissue. MetS—control group of rats with mutation in the leptin receptor gene; MetS + 100 mg and MetS + 200 mg groups constituted animals with mutation and receiving polyphenol extract from pomegranate fruit peels at a dose of 100 mg/kg or 200 mg/kg, respectively; H + 100 mg and H + 200 mg—healthy animals without metabolic syndrome receiving polyphenol extract at a dose of 100 mg/kg or 200 mg/kg. *n* = 6; a = *p* = 0.0531. One-way ANOVA was used for comparison of MetS vs. MetS + 100 mg vs. MetS + 200 mg, and a t-test was used for MetS + 100 mg vs. H + 100 mg and for MetS + 200 mg vs. H + 200 mg comparisons.

The superoxide dismutase (SOD) activity was higher in the MetS group compared to groups with PPE administration (Figure 3). However, neither the 100 mg/kg nor the 200 mg/kg dose significantly affected SOD activity compared to healthy objects (Figure 3). This indicates that the PPE extract significantly reduces SOD activity. As expected, the ROS/RNS level shows tendency (*p* = 0.0531) to higher levels in the control group (MetS) compared to groups with PPE administration (Figure 4). There was no statistical difference in the ROS/RNS level between MetS groups and healthy ones at the same PPE dose (Figure 4). We observed a decreased ROS/RNS level in MetS supplemented with PPE, but the lack of statistical significance was due to the low *n* number.

**Figure 5:**
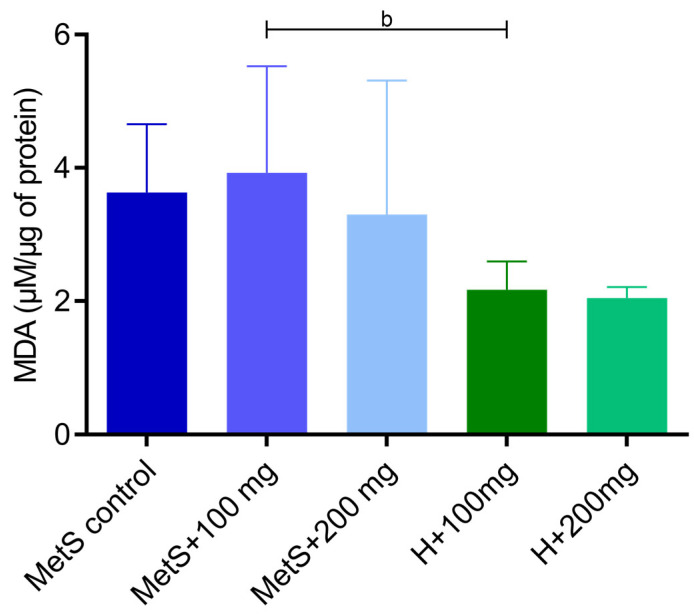
Malondialdehyde (MDA) concentration in splenic tissue. MetS—control group of rats with mutation in the leptin receptor gene; MetS + 100 mg and MetS + 200 mg groups constituted animals with mutation and receiving polyphenol extract from pomegranate fruit peels at a dose of 100 mg/kg or 200 mg/kg, respectively; H + 100 mg and H + 200 mg—healthy animals without metabolic syndrome receiving polyphenol extract at a dose of 100 mg/kg or 200 mg/kg. b = *p* < 0.05. *n* = 6; one-way ANOVA was used for comparison of MetS vs. MetS + 100 mg vs. MetS + 200 mg, and a t-test was used for MetS + 100 mg vs. H + 100 mg and for MetS + 200 mg vs. H + 200 mg comparisons.

The administration of PPE showed no effect on lipid oxidation level in rats with metabolic syndrome (Figure 5). However, the malondialdehyde (MDA) level was lower in healthy subjects compared to the metabolic syndrome group at 100 mg/kg PPE, but the dose of 200 mg/kg PPE abolished this difference (Figure 5).

### 2.2. Apoptosis and Inflammation

**Figure 6:**
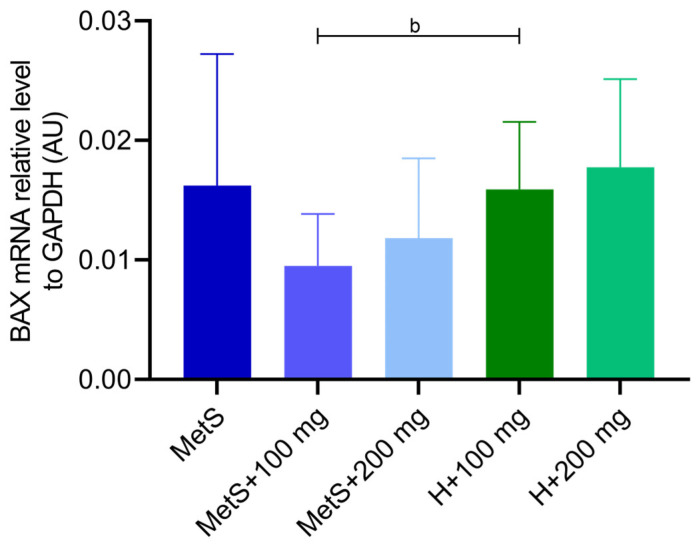
Expression of *BAX* gene in splenic tissue. MetS—control group of rats with mutation in the leptin receptor gene; MetS + 100 mg and MetS + 200 mg groups constituted animals with mutation and receiving polyphenol extract from pomegranate fruit peels at a dose of 100 mg/kg or 200 mg/kg, respectively; H + 100 mg and H + 200 mg—healthy animals without metabolic syndrome receiving polyphenol extract at a dose of 100 mg/kg or 200 mg/kg. *n* = 6; b = *p* = 0.0525. One-way ANOVA was used for comparison of MetS vs. MetS + 100 mg vs. MetS + 200 mg, and a t-test was used for MetS + 100 mg vs. H + 100 mg and for MetS + 200 mg vs. H + 200 mg comparisons.

**Figure 7:**
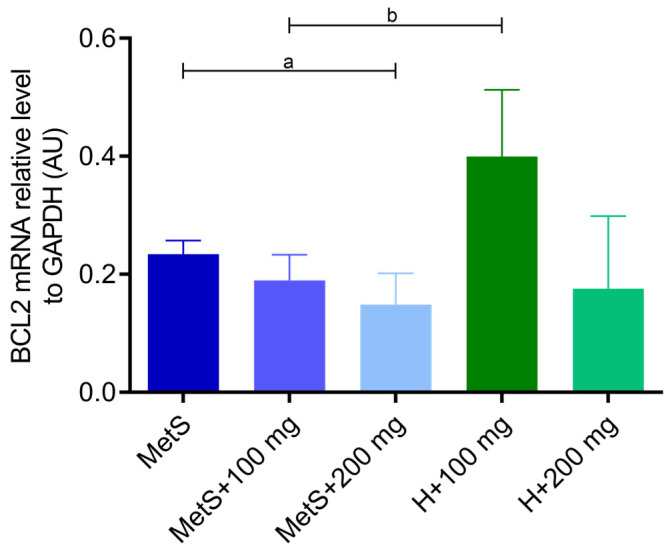
Expression of *BCL-2* gene in splenic tissue. MetS—control group of rats with mutation in the leptin receptor gene; MetS + 100 mg and MetS + 200 mg groups constituted animals with mutation and receiving polyphenol extract from pomegranate fruit peels at a dose of 100 mg/kg or 200 mg/kg, respectively; H + 100 mg and H + 200 mg—healthy animals without metabolic syndrome receiving polyphenol extract at a dose of 100 mg/kg or 200 mg/kg. a = *p* < 0.05, b = *p* < 0.01. *n* = 6; one-way ANOVA followed by Tukey’s test was used for comparison of MetS vs. MetS + 100 mg vs. MetS + 200 mg, and a t-test was used for MetS + 100 mg vs. H + 100 mg and for MetS + 200 mg vs. H + 200 mg comparisons.

The administration of PPE showed no impact on the expression of the proapoptotic *BAX* gene (Figure 6). However, the expression of the antiapoptotic *BCL-2* gene was lower in the MetS + 200 mg group compared to the MetS group (Figure 7). The expression of the *BCL-2* gene was also lower in the MetS group compared to the healthy group at the same PPE dose of 100 mg/kg (Figure 7), and a similar tendency was observed in *BAX* expression (*p* = 0.0525, Figure 6). The dose of 200 mg/kg PPE eliminated this effect (Figure 6 and Figure 7).

**Figure 8:**
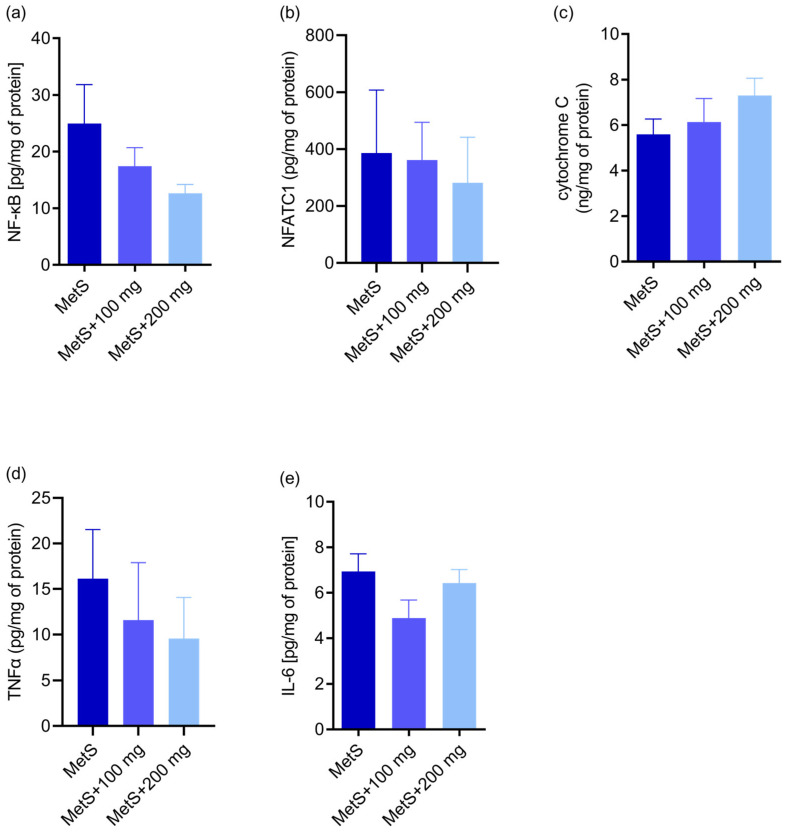
Concentration of pro-apoptotic factor NF-κB (*p* = 0.18; (**a**)); pro-inflammatory NFATc1 (*p* = 0.59; (**b**)); cytochrome c (*p* = 0.38; (**c**)); TNFα (*p* = 0.19; (**d**)); IL-6 concentration (*p* = 0.15; (**e**)) in metabolic syndrome groups in splenic tissue. MetS—control group of rats with mutation in the leptin receptor gene; MetS + 100 mg and MetS + 200 mg groups constituted animals with mutation and receiving polyphenol extract from pomegranate fruit peels at a dose of 100 mg/kg or 200 mg/kg, respectively. *n* = 6; one-way ANOVA was used.

In the MetS groups, there were no differences in the concentration of proapoptotic factor NF-κB (*p* = 0.18; Figure 8a) and pro-inflammatory NFATc1 (*p* = 0.59; Figure 8b). Moreover, there was no impact of PPE on the cytochrome c synthesis (*p* = 0.38; Figure 8c) in metabolic syndrome, and the TNFα (*p* = 0.19; Figure 8d) and the IL-6 concentrations (*p* = 0.15; Figure 8e) showed no differences depending on PPE administration in the metabolic syndrome groups.

### 2.3. MMP-9 and MMP-2 Activity and MMP-2 Concentration

**Figure 9:**
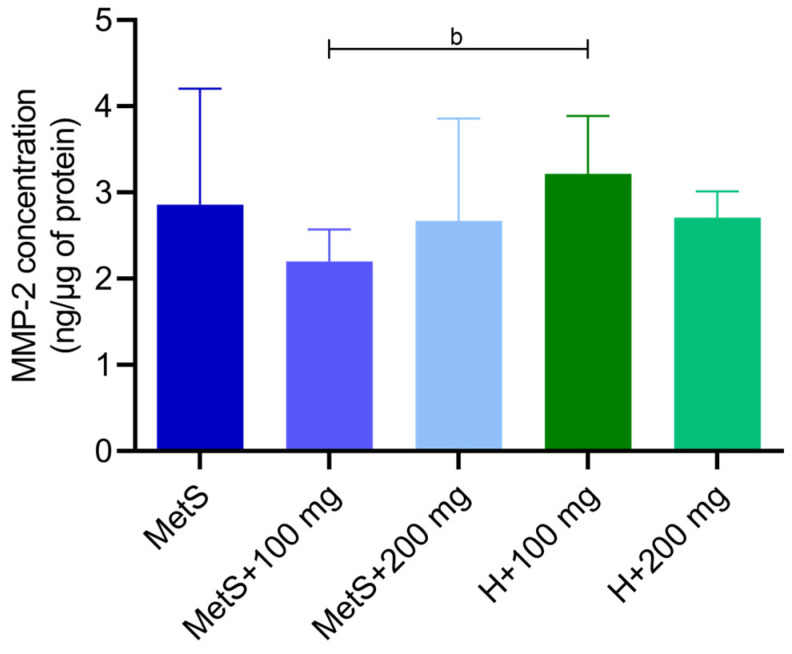
Matrix metalloproteinase 2 (MMP-2) concentration in splenic tissue. MetS—control group of rats with mutation in the leptin receptor gene; MetS + 100 mg and MetS + 200 mg groups constituted animals with mutation and receiving polyphenol extract from pomegranate fruit peels at a dose of 100 mg/kg or 200 mg/kg, respectively; H + 100 mg and H + 200 mg—healthy animals without metabolic syndrome receiving polyphenol extract at a dose of 100 mg/kg or 200 mg/kg. b = *p* < 0.01. *n* = 6; one-way ANOVA was used for comparison of MetS vs. MetS + 100 mg vs. MetS + 200 mg, and a t-test was used for MetS + 100 mg vs. H + 100 mg and for MetS + 200 mg vs. H + 200 mg comparisons.

**Figure 10:**
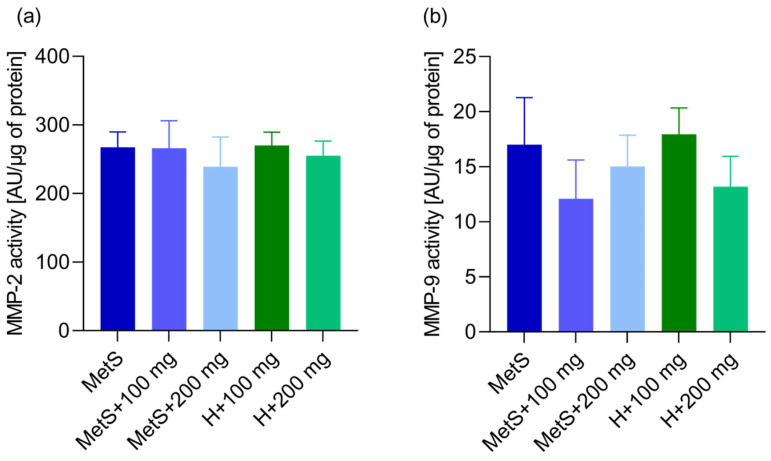
MMP-2 (**a**) and MMP-9 (**b**) activity in splenic tissue. MetS—control group of rats with mutation in the leptin receptor gene; MetS + 100 mg and MetS + 200 mg groups constituted animals with mutation and receiving polyphenol extract from pomegranate fruit peels at a dose of 100 mg/kg or 200 mg/kg, respectively; H + 100 mg and H + 200 mg—healthy animals without metabolic syndrome receiving polyphenol extract at a dose of 100 mg/kg or 200 mg/kg. *n* = 6; one-way ANOVA was used for comparison of MetS vs. MetS + 100 mg vs. MetS + 200 mg, and a t-test was used for MetS + 100 mg vs. H + 100 mg and for MetS + 200 mg vs. H + 200 mg comparisons.

There was no effect of the PPE on the matrix metalloproteinase 2 (MMP-2) concentration in the metabolic syndrome groups (Figure 9). A lower concentration of MMP-2 was found in the MetS + 100 mg group compared to the healthy group H + 100 mg (Figure 9). There was no effect of the PPE on MMP-2 and MMP-9 activity (Figure 10).

## 3. Discussion

In recent decades, researchers have demonstrated the antioxidant, anti-inflammatory, antibacterial, and analgesic effects of flavonoids [10]. Polyphenols can influence antioxidant enzyme systems, restore mitochondrial function, and regulate antioxidant responsive signaling pathways [34]. The growing interest in natural phenolic compounds of plant origin has resulted in significant development of research looking for new properties of polyphenols and their impact on living organisms. One of the rich sources of natural phenolic compounds is *Punica granatum* L. Pomegranate peel extract has high antioxidant activity and can be an excellent source of natural antioxidants [16,35,36]. Medjakovic and Jungbauer reviewed that pomegranates can be used for the treatment of obesity and diabetes and also helps in regulating blood lipid levels, thus alleviating metabolic syndrome. By activating peroxisome proliferator-activated receptors, pomegranate may contribute to the reduction in serum triglycerides and glucose, improvement in insulin sensitivity, and increase in high-density lipoprotein [37]. In this study, we assessed the effects of pomegranate extract intake on oxidative stress, immune response, and apoptosis in the spleens of rats with metabolic syndrome.

Catalase and glutathione peroxidase play pivotal roles in preventing oxidative damage to cells by degrading hydrogen peroxide [38,39]. We showed that the MetS + 100 mg group had significantly lower enzymes activities than the healthy group treated with PPE at a dose of 100 mg/kg. It confirms that metabolic syndrome reduces the antioxidant activity in the body. Furthermore, we demonstrated that CAT and GPX activities in spleen tissue were higher in the MetS group receiving 200 mg/kg PPE compared with the 100 mg/kg group, suggesting that PPE enhanced the antioxidant response at the 200 mg/kg dose. The MetS + 100 mg group had the lowest antioxidant activity among other groups. PPE treatment at a dose of 200 mg/kg showed no difference between the healthy group and the MetS group. Our results indicate that *Punica granatum* affects GPX and CAT activity and a dose of 200 mg/kg of pomegranate extract increases antioxidant activity to the level observed in healthy rats receiving PPE. In the same experimental model, Radajewska and Szyller et al. showed that the groups treated with pomegranate extract had also higher CAT activity in kidney tissue [40]. The concentration of oxidized glutathione was higher in the spleen of obese mice [41]. In the spleen of chickens infected with *Mycoplasma gallisepticum*, the CAT and GPX values were reduced compared to healthy and infected subjects treated with another flavonoid—baicalin (450 mg/kg). Baicalin treatment alleviated oxidative stress and restored normal CAT and GPX levels in the spleen of infected chickens [1].

Superoxide dismutase is another important component of the antioxidative system. The protective role of SOD is to catalyze the dismutation of superoxide anion to hydrogen peroxide [42]. An imbalance between antioxidants and oxidants results in oxidative stress. In metabolic syndrome, oxidative stress is one of the main factors contributing to the development of a number of complications, including cardiovascular problems [43,44,45]. Punicalagin, a phenolic compound found in pomegranate extract, exhibits antioxidant properties by scavenging free radicals [46]. We found that ROS/RNS concentration was lower in the spleen of MetS rats receiving PPE (*p* = 0.0531). The reduction in ROS/RNS levels during PPE treatment may explain the reduction in SOD activity in the MetS groups with PPE administration. In the spleen of the MetS control group, SOD activity was the highest. In the kidneys of rats with MetS, the level of ROS/RNS was higher compared to the group supplemented with PPE at a dose of 100 mg/kg. However, contrary to our findings, a higher dose of PPE corresponded to a higher ROS/RNS concentration than the lower dose of pomegranate extract [40]. Resveratrol, a phenolic compound naturally found in fruits, restored the activity of SOD, GPX, and CAT in a mouse model of cigarette smoke-induced lung injury [47]. Resveratrol has been shown to reduce oxidative stress in the liver of prediabetic rats. An increase in TBARS concentration and a decrease in SOD activity were found in the liver of rats on a high-fructose diet [48]. Chang et al. showed that resveratrol supplementation significantly alleviates oxidative stress by reducing superoxide anion and increasing SOD protein expression in the spleen tissues of diabetic rats [49]. Reduced SOD activity is suggested to be associated with metabolic syndrome as well as impaired insulin sensitivity and β-cell dysfunction [50]. Furukawa et al. showed that increased oxidative stress in adipose tissue is a factor initiating the metabolic syndrome [51]. The progression of oxidative stress and mitochondrial dysfunction is caused by the excessive formation of free fatty acids [52]. During lipid peroxidation, free radicals alter the double carbon bonds in lipids, leading to the formation of lipid peroxide radicals, hydroperoxides, and MDA production [53]. Fuhrman et al. showed that pomegranate juice reduces the cellular uptake of oxidized LDL (ox-LDL) and inhibits cellular cholesterol metabolism, resulting in reduced foam cell formation [54]. Song et al. demonstrated that a diet rich in walnuts and chokeberry (containing antioxidant phenolic compounds) reduced serum and liver MDA levels to the control group levels in d-galactose-induced mouse aging model [55]. Gheorghe et al. demonstrated a higher degree of lipid peroxidation in the spleens of obese mice [41]. In our study, we did not confirm the effect of PPE on lipid oxidation in metabolic syndrome; however, there was a tendency towards higher MDA levels in the MetS groups compared to healthy subjects. We found that the 200 mg/kg PPE dose slightly reduced the MDA levels, with a difference between the MetS + 100 mg and H + 100 mg groups, whereas there was no difference between the same groups receiving 200 mg/kg PPE. Amri et al. evaluated the effects of pomegranate seed oil, leaves, juice, and peel on brain oxidative stress and lipid profile in high-fat, high-fructose diet-induced obese rat. They showed that the administration of these extracts reduced the MDA levels and increased the SOD and GPX levels, confirming the neuroprotective effect of pomegranate [56]. Moreover, in alloxan-induced diabetes rat models, pomegranate peel extracts reduced serum MDA levels [57]. Furthermore, in people with type 2 diabetes, treatment with pomegranate juice and pomegranate peel extract also reduced the level of lipid peroxidation [58,59,60]. Data from the literature confirm the beneficial effect of pomegranate extracts on lipoprotein oxidation, which is not clearly confirmed by the results of our research. 

In obesity-related disorders, high oxidative stress correlates with long-term inflammation [44]. Since the spleen is the largest lymphoid organ, it appears to play one of the key roles in maintaining the redox balance and modulation of inflammation [61]. During LPS-induced inflammation, the spleen starts to produce some proteins only locally; hence, the inflammatory proteome of the spleen microenvironment is different from that observed in the plasma [62]. Chronic low-grade inflammation caused by obesity leads to metabolic disorders responsible for dysfunction of many organs [52,63,64]. Oxidative stress can lead to the activation of transcription factors, e.g., nuclear factor kappa B (NF-κB) [52]. NF-κB promotes the expression of genes mediating cell proliferation and the release of antimicrobial molecules and cytokines and is considered as a proapoptotic factor. Moreover, NF-κB links the metabolic and inflammatory responses [65]. However, the NF-κB pathway may exhibit both anti- and pro-oxidant properties during oxidative stress [66]. Nuclear factors of activated T cells (NFAT) are also involved in the regulation of pro-inflammatory factors in immune cells. NFATc3 plays a key role in adipose tissue inflammation and insulin resistance in obesity by activating pro-inflammatory genes [67]. Here, no effect of PPE administration on the concentration of proapoptotic NF-κB and proinflammatory NFATc1 in the metabolic syndrome groups was observed; moreover, PPE did not affect the synthesis of cytochrome c and the concentrations of TNF-α and IL-6 in the spleen tissue. In diabetic rats, resveratrol treatment decreased the expression of NF-κB p65 but increased the levels of TNFα and IL-6 in spleen tissues [49].

Metabolic syndrome is associated with increased splenocyte apoptosis, which may result in immune suppression, and apoptotic splenocytes may induce immune tolerance and impaired response to infection [68]. The BCL-2 family proteins include antiapoptotic regulators (including BCL-2, BCL-XL, and BCL-W) and proapoptotic regulators (e.g., BAX, BAK, and BOK) [69,70]. In obese Zucker-fa/fa rats, *BCL-2* mRNA levels were lower than in control rats, and *BAX* mRNA increased with the development of obesity. In obese rats, the BCL-2/BAX protein-to-mRNA ratio was lower in brown adipocytes [71]. In our study, *BCL-2* gene expression was lower in the MetS group compared to the healthy group when both received 100 mg/kg PPE, which seems to confirm the proapoptotic effect of MS. A dose of 200 mg/kg PPE caused a decrease in *BCL-2* expression in both groups. Moreover, the groups treated with 200 mg PPE tended to have the lowest *BCL-2* expression. In turn, *BAX* expression showed no differences in the MetS groups, we observed a tendency toward lower expression in the MetS group + 100 mg compared to H + 100 mg, and the 200 mg PPE dose did not show this effect. It is worth emphasizing that the tendency to reduce *BAX* expression in the MetS group may result from the impairment of the BCL-2 family pathway and the imbalance of pro- and antiapoptotic factors at the gene level, which may be caused by MS itself. In adipocytes with palmitate-induced apoptosis, pomegranate flower extract decreased the expression of the *BAX* gene and increased the expression of the *BCL-2* gene, demonstrating antiapoptotic effects [72]. Zhao et al. showed that the combination of exercise and pomegranate extract attenuated abnormalities in spleen histomorphology and inhibited apoptosis in splenocytes in obese rats [73]. On the other hand, a study evaluating the antiproliferative potential of pomegranate seed and peel extracts against a liver cancer cell line showed increased expression of the *BAX* gene and decreased expression of the *BCL-2* gene [74]. Similar effects have been observed in breast cancer lines and in UVA-induced changes in cell proliferation, as discussed in more detail in the review by Sharma [75,76,77].

Matrix metalloproteinases (MMPs) are zinc-dependent proteolytic enzymes. MMPs are mainly involved in the degradation of the extracellular matrix and tissue remodeling. MMPs are crucial for cell proliferation, migration and differentiation, tissue repair, and angiogenesis, but are also involved in cell apoptosis. Changes in the expression and activity of MMPs occur in pathological conditions, including atherosclerosis, obesity, or inflammation [78,79]. MMP-2 gene variants may be associated with the risk of developing MetS. The serum MMP-2 concentration was higher in patients with MetS and correlated with the clinical parameters of MetS [80,81]. Increased expression or activity of MMP-2 and/or MMP-9 proteins has been most frequently observed in dysglycemia, hypertension, oxidized LDL and high LDL levels, and inflammation [82,83]. On the other hand, Goncalves et al. showed no differences in the pro-MMP-2 levels in patients with MetS and healthy individuals [84], while Kosmala et al. demonstrated decreased MMP-2 plasma levels in young obese women [85]. Miksztowicz et al. showed that induced insulin resistance reduces MMP-2 and MMP-9 activity in adipose tissue, concluding that it is not a source of MMP-2 and MMP-9 in circulation [86]. We obtained inconsistent results regarding the concentration of MMP-2 in spleen tissue. A lower concentration of MMP-2 was found in the MetS group supplemented with 100 mg/kg PPE compared to the healthy group receiving the same supplementation; however, the dose of 200 mg/kg showed no differences between the MetS group and the healthy group. We found no differences between the MetS groups receiving the PPE treatment, indicating that the extract had no effect on MMP-2 concentration. Furthermore, we found no differences in the activity of MMP-2 and MMP-9.

This study has some limitations. The study groups were characterized by a relatively small number of subjects (*n* = 6). It is worth emphasizing that improving the welfare of animals used in the experiments requires reducing the number of objects, which refers to the 3Rs principle in animal experiments (replacement, reduction, and refinement). In the future, an expansion of the research should be considered to include further doses of the extract and include a group of healthy subjects not receiving the extract; however, in the context of our results, it was crucial to compare all groups with metabolic syndrome. The composition of EPP is not standardized, and the content of phenols (quantitative and qualitative) depends on many factors, including the variety of fruit; its ripeness; and the storage conditions, which is also a kind of limitation. Of course, double-blind randomized controlled trials are necessary to prove the bioavailability and efficacy of *Punica granatum* L. peel in humans.

In conclusion, pomegranate peel extract affects the oxidative state of the spleen in the course of metabolic syndrome. The use of a 200 mg/kg dose of the extract leads to an increase in the activity of catalase and glutathione peroxidase, as the basic enzymes in the antioxidative system. Moreover, since pomegranate reduces the ROS/RNS level, leading to the further normalization of SOD antioxidant, by examining the pathomechanism, we showed a tendency for increased lipid peroxidation in the course of the metabolic syndrome, although only selected concentrations of PPE reduced the degree of peroxidation, which requires further research. We also checked whether metabolic syndrome and PPE regulate the inflammatory response and showed that the mechanism of action of pomegranate does not underlie NF-κB-dependent induction of inflammation. We also confirm that metabolic syndrome can affect the balance between pro- and antiapoptotic factors of the BCL-2 family pathway, favoring proapoptotic activity.

## 4. Materials and Methods

### 4.1. Study Group

**Figure 11:**
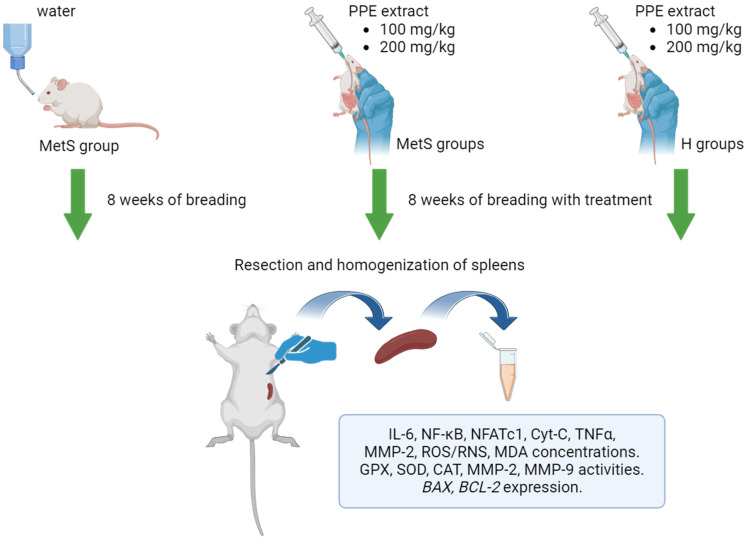
Scheme of the experiment protocol. MetS—control group of rats with mutation in the leptin receptor gene; MetS + 100 mg and MetS + 200 mg groups constituted animals with mutation and receiving polyphenol extract from pomegranate fruit peels at a dose of 100 mg/kg or 200 mg/kg, respectively; H + 100 mg and H + 200 mg—healthy animals without metabolic syndrome receiving polyphenol extract at a dose of 100 mg/kg or 200 mg/kg. Created with BioRender.com. https://www.biorender.com/ accessed on 27 September 2024.

Adult male Zucker Diabetic Fatty (ZDF-Leprfa/Crl, fa/fa) rats and their healthy counterparts (fa/+) were used in this study. The rats were obtained from Sulzfeld (Charles River Laboratories, Research Models and Services, Germany GmbH). The animals were housed in cages (two rats/cage) and kept under controlled temperatures (22 ± 2°C), humidity (55 ± 5%), and light/dark (12/12 h) cycles. All procedures on experimental animals were performed in accordance with the Guide for the Care and Use of Experimental Animals published by the Ministry of Science and Higher Education in Poland [87]. Ad libitum access to the same diet was provided (Purina LabDiet 5008, Charles River Laboratories, Arden Hills, MN, USA). After two weeks of acclimatization, the rats were divided into five study groups of six animals each. Before our studies, a minimum sample size was calculated in terms of the values analyzed to ensure statistically significant data. The control group (MetS) consisted of rats with mutation in the leptin receptor gene (ZDF fa/fa). Two study groups consisted of ZDF fa/fa rats receiving polyphenol extract from pomegranate fruit peel (PPE) at a dose of 100 mg (MetS + 100 mg) or 200 mg/kg (MetS + 200 mg), and two groups consisted of animals without metabolic syndrome (fa/+) also receiving the polyphenol extract at a dose of 100 mg (H + 100 mg) or 200 mg/kg (H + 200 mg The PPE was administered with water through a gastric tube. The procedure lasted 8 weeks. At the end of housing, the body weight of the rats reached 407.1 ± 32.7 g in the MetS control group, 382.1 ± 35.5 g in the MetS + 100 mg group, 392.8 ± 36.3 g in the Mets + 200 mg group, 359 ± 12.9 g in the H + 100 mg group, and 321 ± 60.5 g in the H + 200 mg group. Body weight increased in all groups, but no statistically significant differences were confirmed between groups. Glucose concentration in rats with metabolic syndrome (MetS, MetS + 100 mg, and MetS + 200 mg) was significantly higher than in rats without the syndrome (H + 100 mg and H + 200 mg); a similar pattern was also observed in the lipid profile [88]. The experimental procedure is shown in Figure 11. The investigation was approved by the Ethics Committee for Experiments on Animals at the Ludwik Hirszfeld Institute of Immunology and Experimental Therapy Polish Academy of Sciences, Wroclaw, Poland (resolution no. 53/2017 of 17 May 2017).

### 4.2. Preparation of Polyphenol Extract

A detailed description of the preparation of pomegranate peel extract followed the protocol published by Radajewska and Szyller et al. and Niewiadomska et al. [19,40]. Briefly, *Punica granatum* L. peels (cultivar Mollar de Elche, Alicante, Spain) were used to prepare the extract. The dried peels were extracted and re-extracted with 50% ethanol (twice). The extract was then concentrated by evaporation at 40°C and next adsorbed using resin. After evaporating the ethanol, the collected fraction was dried in SPT-200 vacuum oven (Zeamil, Krakow, Poland). The content of phenolic compounds in PPE is shown in Table 1.

### 4.3. Spleen Isolation

Before spleen resection, the rats were anesthetized with an intramuscular injection of ketamine (60 mg/kg; Biowet, Puławy, Poland) and medetomidine (0.3 mg/kg; Orion Corporation, Espoo, Finland) and euthanized with an intraperitoneal injection of pentobarbital (0.5 mL/kg i.p.; Biowet, Puławy, Poland). The spleens were immediately excised and rinsed with saline, then frozen in liquid nitrogen, and stored in −80°C until homogenization.

### 4.4. Preparation of Tissue Homogenates

Spleens were frozen in liquid nitrogen for several minutes and grinded/crushed with a porcelain mortar and pestle. An aliquot of tissue powder was mixed with a homogenization buffer (50 mmol/L Tris-HCl, 150 mmol/L NaCl, 0.1% Triton X-100, pH 7.4) containing a Protease Inhibitors Cocktail Set III (Sigma-Aldrich, Saint Louis, MO, USA) in ratio 1:5 (w:v). Next, the samples were heated three times to 37°C, frozen in liquid nitrogen, and homogenized on ice using a Pellet Pestle^®^ Motor (Kimble Kontes, Vineland, NJ, USA). The samples were centrifuged (5 min, 17 530 g, 4°C), and the protein concentration in the supernatants was measured using Bradford Protein Assay (Bio-Rad, Hercules, CA, USA). The supernatants and the remaining tissue powder were stored at −80°C until further examination.

### 4.5. RNA Isolation and qRT-PCR

A two-phase RNA isolation was performed using Trizol Reagent (ThermoFisher Scientific, Waltham, MA, USA) according to the manufacturer’s instructions. Briefly, 30 mg of tissue powder was mixed with Trizol and homogenized using a Pellet Pestle^®^ Motor (Kimble Kontes, Vineland, NJ, USA). RNA was extracted with chloroform (Stanlab, Lublin, Poland); then, RNA from the aqueous phase was precipitated with isopropanol (Chempur, Piekary Śląskie, Poland) and washed with 75% ethanol (Chempur, Piekary Śląskie, Poland). The dried RNA pellet was dissolved in UltraPure DEPC-Treated water (ThermoFisher Scientific, Waltham, MA, USA), and the RNA concentration was measured using a NanoDrop Lite Spectrophotometer (ThermoFisher Scientific, Waltham, MA, USA). Reverse transcription was performed on 1000 ng of RNA from each sample using iScript™ cDNA Synthesis Kit (BioRad, Hercules, CA, USA) according to the manufacturer’s instructions. Real-time PCR was performed on 100 ng of cDNA template in duplicate for each gene using iTaq™ Universal SYBR^®^ Green Supermix (BioRad, Hercules, CA, USA) following the manufacturer’s protocol. Reverse transcription and Real-time PCR were performed using a CFX96 Touch Real-Time PCR Detection System (BioRad, Hercules, CA, USA). Bcl-2-associated X protein (*BAX*) and B-cell lymphoma-2 (*BCL2*) gene expression was assessed (the 5′–3′ sequences of the primers are shown in Table 2). The glyceraldehyde 3-phosphate dehydrogenase (*GAPDH*) gene was used as a housekeeping gene, and the calculations were performed using the delta-delta Ct method. The amplicon lengths were 102 bp for *BCL2*, 566 bp for *BAX*, and 248 bp for *GAPDH*. 

### 4.6. Enzyme-Linked Immunosorbent Assays

The concentrations of interleukin 6 (IL-6), nuclear factor kappa B (NF-κB), nuclear factor of activated T-cells, cytoplasmic 1 (NFATc1), cytochrome C (Cyt-C), tumor necrosis factor α (TNFα), and matrix metalloproteinase 2 (MMP-2) in the tissue homogenates were measured using ELISA kits. All procedures were performed according to the manufacturers’ protocols. The Rat IL-6 ELISA Kit cat. no. CSB-E04640r (Cusabio, Huston, TX, USA), the Rat NF- κB ELISA Kit cat. no. CSB-E13148r (Cusabio, Huston, TX, USA), the Rat NFATC1 ELISA Kit cat. no. EKF58951 (Biomatik, Wilmington, DE, USA), the Rat Cyt-C ELISA KIT cat. no. ERO893 (FineTest, Wuhan, China), the Rat TNFα ELISA kit cat. no. EK0526 (Boster, Pleasanton, CA, USA), and the Total MMP-2 Quantikine^®^ ELISA kit cat. no. MMP200 (R&D Systems, Minneapolis, MN, USA) were used. Absorbance was measured using a microplate reader (Tecan Spark Microplate Multimode Plate Reader, Tecan Trading AG, Männedorf, Switzerland).

### 4.7. Glutathione Peroxidase Activity

Glutathione Peroxidase Assay Kit cat. no 703102 (Cayman Chemical Company, Ann Arbor, MI, USA) was used to determine the GPX activity. The test was performed in accordance with the manufacturer’s instructions. Briefly, GPX catalyzes the reduction in hydroperoxide; then, the product of this reaction—oxidized glutathione—is reduced during the reaction with NADPH. Oxidation of NADPH to NADP+ causes a decrease in absorbance at 340 nm. The change in absorbance was measured at 340 nm with a microplate reader (Tecan Spark Microplate Multimode Plate Reader, Tecan Trading AG, Männedorf, Switzerland).

### 4.8. Lipid Oxidation Level

TBARS Assay Kit cat. no 10009055 (Cayman Chemical Company, Ann Arbor, MI, USA) was used to assess the lipid oxidation level. The test was performed in accordance with the manufacturer’s instructions. The measurement of thiobarbituric acid reactive substances (TBARSs) is used to assess lipid peroxidation. Malondialdehyde (MDA) is a natural product of lipid peroxidation. MDA reacts with thiobarbituric acid (TBA) at 90–100°C and under acidic conditions. The MDA-TBA adduct is measured colorimetrically at 530–540 nm. Absorbance was measured at 535 nm with a microplate reader (Tecan Spark Microplate Multimode Plate Reader, Tecan Trading AG, Männedorf, Switzerland).

### 4.9. Superoxide Dismutase Activity

Superoxide dismutase (SOD) Assay Kit cat. no 19160-1KT-F (Sigma-Aldrich, Saint Louis, MO, USA) was used to measure the SOD activity. The test was performed in accordance with the manufacturer’s instructions. Briefly, the WST-1 (2-(4-Iodophenyl)-3-(4-nitrophenyl)-5-(2,4-disulfophenyl)-2H-tetrazolium, monosodium salt)—water-soluble tetrazolium salt after reduction with superoxide anion—produces a water-soluble formazan dye. The reduction is inhibited by SOD; then, the SOD inhibition activity is quantified by measuring the decrease in color change at 440 nm. Absorbance was measured with a microplate reader (Tecan Spark Microplate Multimode Plate Reader, Tecan Trading AG, Männedorf, Switzerland).

### 4.10. ROS/RNS

OxiSelectTM In Vitro ROS/RNS Assay Kit cat. no. STA-347 (Cell Biolabs, INC., San Diego, CA, USA) was used to assess the total ROS/RNS level including hydrogen peroxide, nitric oxide, peroxyl radical, and peroxynitrite anion. The test was performed in accordance with the manufacturer’s instructions. The assay uses a specific ROS/RNS fluorogenic probe, dichlorodihydrofluorescin DiOxyQ (DCFH-DiOxyQ). The probe is first primed with a quench removal reagent and then stabilized in the reactive DCFH form, which reacts with ROS and RNS species. The DCFH is rapidly oxidized to the highly fluorescent 2′, 7′-dichlorodihydrofluorescein (DCF) and the fluorescence intensity is proportional to the total ROS/RNS levels. Fluorescence was measured at an excitation wavelength of 480 nm and an emission wavelength of 530 nm using a microplate reader (Tecan Spark Microplate Multimode Plate Reader, Tecan Trading AG, Männedorf, Switzerland).

### 4.11. Catalase Activity

Catalase (CAT) Assay Kit cat. no 707002 (Cayman Chemical Company, Ann Arbor, MI, USA) was used to measure the catalase activity. The test was performed in accordance with the manufacturer’s instructions. Catalase reacts with methanol in the presence of H_2_O_2_ to form formaldehyde. The produced formaldehyde reacts with chromogen—Purpald (4-amino-3-hydrazino-5-mercapto-1,2,4-triazole)—causing a color change. Absorbance was measured at 540 nm with a microplate reader (Tecan Spark Microplate Multimode Plate Reader, Tecan Trading AG, Männedorf, Switzerland).

### 4.12. Zymography

The gelatinolityc activity of MMPs was assessed using gelatin zymography. Briefly, the first step of zymography was electrophoresis using 7,5% SDS-PAGE with copolymerized gelatin. Electrophoresis was performed in Mini-Protean II (BioRad, Hercules, CA, USA). After electrophoresis, the gels were washed three times with 2,5 % Triton X-100 to remove SDS and then incubated with an enzyme assay buffer (0.05 M Tris-HCl, pH 7.5 containing 5 mM CaCl2, 0.2 M NaCl, 0.05% NaN3) at 37°C for 18 h. After incubation, the gels were stained with 0.3% CBB R-250 (MP Biomedicals, Illkirch-Graffenstaden, France) mixed with 0.2% CBB G-250 (MP Biomedicals, Illkirch-Graffenstaden, France) and then destained until white bands were visible on a blue background. To measure MMP activity, zymograms were scanned with a densitometer (BioRad G5-800, Hercules, CA, USA) and analyzed using Quantity One software v. 4.6.9 (BioRad, Hercules, CA, USA). The HT10-80 cell line was used as a positive control, and the relative MMPs activity was expressed in arbitrary units (AU) per µg of protein in the sample.

### 4.13. Statistical Analysis

Statistical analysis was performed using GraphPad Prism v. 8.0.1 (GraphPad Software, Boston, MA, USA). The Shapiro–Wilk test was used to assess the normality of data distribution. Depending on the normality of the data distribution, the t-test or the Mann–Whitney U test was performed when comparing MetS + 100 mg vs. H + 100 mg and MetS + 200 mg vs. H + 200 mg. In the case of MetS vs. MetS + 100 mg vs. MetS + 200 mg, the Kruskal–Wallis test or a one-way ANOVA analysis was used, followed by Dunn’s or Tukey’s tests, respectively. The results are presented as mean ± SD. The results were considered statistically significant for *p* values of 0.05 or less.

## Figures and Tables

**Table 1:** Mass spectrum characteristic and content of phenolic compounds in pomegranate peel extract [26].

Rt	MS [M-H]^−^ (*m/z*)	MS/MS [M-H]^−^ (*m/z*)	Name of Compounds	Polyphenols Content
1.67	331	271/169	Galloyl-glucose	2.00 ± 0.03
1.73	781	721/601	Punicalin α/A	3.11 ± 0.06
2.02	1083	611/331/146	HHDP-galloyl-hexoside (punicalagin)	4.20 ± 0.09
2.12	1083	781/622/301	Punicalagin isomer	14.82 ± 1.04
2.33	933	631/450/301	Ellagitannin	4.71 ± 0.40
2.87	1083	781/301	HHDP-gallagyl-hexoside (punicalagin)	93.91 ± 2.05
3.12	1085	907/783/301	Ellagic acid derivative	2.49 ± 0.53
3.69	1083	781/301	HHDP-gallagyl-hexoside (punicalagin)	157.0 ± 2.65
3.89	799	301	Granatin A	4.74 ± 0.32
5.08	783	481/301	Ellagitannin	25.86 ± 1.53
6.20	1085	933/301	Digalloyl-gallagyl-hexoside	10.37 ± 0.65
6.25	783	481/301	Ellagitannin	13.51 ± 0.99
6.38	463	301	Ellagic acid-hexoside	33.63 ± 1.23
6.89	951	907/635/301	Galloyl-HHDP-DHHDP-hex (granatin B)	2.68 ± 0.11
			Total (mg/g dw)	373.05

**Table 2:** The 5′–3′ sequences of the primers used in this study.

Gene (NM Code)	Forward Prime	Reverse Primer
BAX (NM_017059.2)	CACGTCTGCGGGGAGTCA	TAGGAAAGGAGGCCATCCCA
BCL2 (NM_016993.2)	GGTGAACTGGGGGAGGATTG	AGAGCGATGTTGTCCACCAG
GAPDH (NM_017008.4)	AGTGCCAGCCTCGTCTCATA	GATGGTGATGGGTTTCCCGT

## Data Availability

The original contributions presented in the study are included in the article; further inquiries can be directed to the corresponding authors.
